# Preface – 10 ^th^ ISTIB

**DOI:** 10.3897/zookeys.801.31568

**Published:** 2018-12-03

**Authors:** Elisabeth Hornung, Stefano Taiti, Katalin Szlavecz

**Affiliations:** 1 Szent István University, Faculty of Veterinary Science, Budapest, Hungary Szent István University Budapest Hungary; 2 Istituto di Ricerca sugli Ecosistemi Terrestri, CNR, Sesto Fiorentino, Firenze, Italy Istituto di Ricerca sugli Ecosistemi Terrestri Firenze Italy; 3 The John Hopkins University, Baltimore, USA The John Hopkins University Baltimore United States of America

’Even if we have written testimony that the ancient Greek intellectuals were already aware of the existence of woodlice, the history of scientific research on this group of animals starts 2000 years later. … Today isopodological research includes studies on ecology, behavior, nutritional biology, anatomy, functional morphology, neurology, physiology, biogeography, systematics, and phylogenetic analyses based on morphological and molecular data’ (Schmalfuss 2018).

Since the first Symposium on the Biology of Terrestrial Isopods in London, UK (1983), scientists have been meeting every three years to discuss their latest findings on all aspects of terrestrial isopod biology. The 10^th^ symposium, held in Budapest, Hungary in 2017 (http://bio.univet.hu/istib2017/main.html) brought together over 70 participants from 23 countries representing Europe, North and South America, Africa, and Near East. The meeting was organised by the Department of Ecology (University of Veterinary Medicine, Budapest) and by the Hungarian Biological Society with support from the Hungarian Ecological Society, the Hungarian Natural History Museum, the Budapest Zoo and Pensoft Publishers.

Diverse topics at many spatio-temporal scales were presented, all under the umbrella of our beloved crustaceans: woodlice, slaters, pill bugs, sow-bugs, roly-poly-s, landpissebedden, Asseln, porcellini di terra, ászka, pincebogár, мокрици, γουρουνίτσες, δροσομάμουνα, etc. The 30 oral presentations, seven of which were review type invited lectures, and 45 lightning talks connecting to posters, covered research on classical and new fields such as taxonomy, biogeography, molecural biology, agroecosystems, sustainable land use, ecosystem services, climate change, human influence, urbanization, structure, and function. Key words given for the presentations included history, phylogeny, taxonomy (new species), biodiversity, species distribution, ecological biogeography, subterranean occurence, life history, trait approach, habitat fragmentation, parasites, predation, pests, phenology, fluctuating asymmetry, activity, feeding, genetics, ecotoxicology, bioaccumulation, heavy metals, morphology, ultrastructure, physiology, hormones, development, microbiota, symbionts and others.

This ZooKeys special issue is a collection of the presentations of the 10^th^ International Symposium on the Biology of Terrestrial Isopods. The title of the volume, ’Isopods in a Changing World’, reflects the growing interest of the science community and public in the potential responses of biota, including isopods, to global environmental change. In the first part of the volume five overviews summarize our current knowledge, highlight research needs and provide future directions. Two contributions focus on climate change effects from local to global scale. Specifically, the papers dicuss how these effects vary depending on species traits, how these differences might lead to changes in species composition, and how the changing climate might shift distribution boundaries of isopod species. Currently the two major types of land use change are agriculture and urbanization. Two papers discuss the individual, population and community responses of terrestrial isopods to the changing landscape. Based upon their abundance, isopods appear to be a successful group both in agricultural systems and in cities. However, generalist, synanthropic species dominate in both systems, and drastic land conversion may lead to local species extinction. The fifth paper reviews the history of isopods as model animals in ecotoxicology. Although isopods have been used as indicator organisms for decades, given the continuing release of various contaminants to both terrestrial and aquatic environments, the effect of environmental toxins on soil fauna and the role of isopods as indicator organisms for pollution level, remain to be timely. Humans, as a force of nature, are at the center of current environmental change. As a result of human residence and land use change, today less than 25% of Earth’ ice free land can be considered as ’wildlands’ with minimal or no human influence (Ellis and Ramankutty 2008). At local scale human impact can be direct, for instance via drastically disturbing the physical environment and selectively promoting or removing species, or indirect, e.g., changing local hydrology or altering nutrient cycles. Human actions, whether deliberate or inadvertent, profoundly, and often irreversibly affect local species presence and abundance, and may act as selective forces leading to evolutionary change. It is therefore important to recognize that interpreting community composition, including that of terresrial isopods, requires knowledge on site history at various temporal scales.

The remaining 18 research papers cover a broad array of disciplines. Contributions to the fields of taxonomy, faunistics and phylogeny indicate that we are far from fully understanding large scale distributions and evolution of Oniscidea. Understanding distributions and abundances at multiple spatial scales requires knowledge on morphological, physiological and ecological traits. Papers continue exploring water relations of terrestrial isopods, which is a fundamental physiological factor determining their success in terrestrial ecosystems. We are also delighted to see contributions in such less studied topics as isopod-parasite interactions and the importance of stridulatory apparatus in isopod communication. Several oral and poster presentations on cave isopod ecology were given during the symposium. In this special volume one of them reports on growth and reproduction of a cave dwelling species. Cave isopods represent a special group with presumably unique life history characteristics, population dynamics, and evolution. Some troglobitic species might be rare, their habitat threatened, and thus of high conservation value.

We hope that for those just getting started in this field this special issue provides an overview and baseline information. Hopefully, the volume will also stimulate ongoing isopod research taking some projects to new directions.
